# Trends in neonatal intensive care unit admissions by race/ethnicity in the United States, 2008–2018

**DOI:** 10.1038/s41598-021-03183-1

**Published:** 2021-12-10

**Authors:** Youngran Kim, Cecilia Ganduglia-Cazaban, Wenyaw Chan, MinJae Lee, David C. Goodman

**Affiliations:** 1 Department of Neurology, McGovern Medical School, The University of Texas Health Science Center, 6431 Fannin St, Houston, TX 77030 USA; 2 Division of Management, Policy and Community Health, School of Public Health, The University of Texas Health Science Center, Houston, TX USA; 3 Department of Biostatistics and Data Science, School of Public Health, The University of Texas Health Science Center, Houston, TX USA; 4 Division of Biostatistics, Department of Population and Data Sciences, The University of Texas Southwestern Medical Center, Dallas, TX USA; 5 The Dartmouth Institute for Health Policy and Clinical Practice, Hanover, NH USA; 6 Department of Pediatrics, Geisel School of Medicine at Dartmouth, Hanover, NH USA

**Keywords:** Neonatology, Preterm birth

## Abstract

To examine temporal trends of NICU admissions in the U.S. by race/ethnicity, we conducted a retrospective cohort analysis using natality files provided by the National Center for Health Statistics at the U.S. Centers for Disease Control and Prevention. A total of 38,011,843 births in 2008–2018 were included. Crude and risk-adjusted NICU admission rates, overall and stratified by birth weight group, were compared between white, black, and Hispanic infants. Crude NICU admission rates increased from 6.62% (95% CI 6.59–6.65) to 9.07% (95% CI 9.04–9.10) between 2008 and 2018. The largest percentage increase was observed among Hispanic infants (51.4%) compared to white (29.1%) and black (32.4%) infants. Overall risk-adjusted rates differed little by race/ethnicity, but birth weight-stratified analysis revealed that racial/ethnic differences diminished in the very low birth weight (< 1500 g) and moderately low birth weight (1500–2499 g) groups. Overall NICU admission rates increased by 37% from 2008 to 2018, and the increasing trends were observed among all racial and ethnic groups. Diminished racial/ethnic differences in NICU admission rates in very low birth weight infants may reflect improved access to timely appropriate NICU care among high-risk infants through increasing health care coverage coupled with growing NICU supply.

## Introduction

In the past 50 years, remarkable advances in the neonatal intensive care units (NICUs) have improved the survival and reduced the morbidity of premature and sick newborns^1^. Delivery of very low birth weight (VLBW) or very preterm (VPT) infants at hospitals with a Level III/IV NICU is now a standard of care in the United States^2^. As increasing hospital competition motivated hospitals to expand the scope of care and retain high-risk patients, the proliferation of NICU units and beds in a higher proportion of hospitals with maternity services and provision of neonatal intensive care extended beyond regional or academic centers^3–5^. Recent U.S. trends show that NICU admissions have increased, particularly in larger and less premature newborns^6^. Expanding the NICU admitted newborn population to less acutely ill newborns suggests that some NICU utilization may be unnecessary. At the same time, some very premature newborns were still not admitted to Level III/IV NICUs. Furthermore, there is evidence that greater bed supply is associated with high NICU admissions, particularly among low-risk newborns^7–10^.

These raise important concerns regarding access to NICU care. Given that birth outcomes determining the need for NICU care tend to be worse among infants who are non-White^11^, infants born to racial minority could suffer disproportionally more from less-than-optimal NICU care access. For example, VLBW infants usually required to be admitted to a NICU and the percentage of VLBW infants is three times as high among non-Hispanic black infants compared to non-Hispanic white infants (2.92% vs 1.02% in 2018)^11^. Race and ethnicity are important factors when assessing health risks and access to health care. While studies on racial/ethnic differences among children address a wide range of areas^12^, researches on racial/ethnic differences among infants have focused mostly on birth outcomes and infant mortality^13^. In this study, we examined temporal trends of NICU admissions by race/ethnicity in the U.S. for all birth weight ranges at the national level and conducted subgroup analyses birth weight group.

## Methods

### Data source and study population

All methods were carried out in accordance with relevant guidelines and regulations. The study protocol was approved by the Committee for the Protection of Human Subjects (CPHS) at the University of Texas Health Science Center at Houston and the need for informed consent was waived by ethics committee because of the deidentified nature of our data.

This is a population-based retrospective cohort study using restricted natality files provided by the National Center for Health Statistics (NCHS) at the U.S. Centers for Disease Control and Prevention (CDC). All births to mothers whose state of residence was U.S. states and the District of Columbia between January 1, 2008, and December 31, 2018 were included^14^. Since information on NICU admission was exclusive to the 2003 revision of the U.S. birth certificate, we excluded births recorded using the earlier version (12.1%). We also excluded those weighing less than 500 g (0.1%) or born before 23 completed weeks of gestational age (0.1%) as they are generally not considered viable with current technology^15,16^. Finally, we excluded births with unknown NICU information (0.3%), birthweight (0.1%), gestational age (0.1%), or Apgar score (0.4%).

### NICU admission and race/ethnicity

The primary outcome-of-interest was a NICU admission rate, which was measured as the proportion of live births who were admitted to a NICU. According to the CDC’s guideline for completion of the 2003 U.S. birth certificate, NICU admission is defined as “admission into a facility or unit staffed and equipped to provide continuous mechanical ventilator support for a newborn”^17^. This definition of NICU care is comparable to Levels III and IV of neonatal care as established by the American Academy of Pediatrics (AAP)^2^. The primary exposure of interest was maternal race/ethnicity as reported separately on birth certificates. We used the bridged race for responses that included more than one race and combined the bridged race and Hispanic ethnicity into the following categories: non-Hispanic white (“white”), non-Hispanic black (“black”), Hispanic, and other.

### Risk adjustment

We risk adjusted NICU admission rates to account for the differences in the infant health status by race/ethnicity over the study period. Neonatal characteristics indicating an infant’s health status from well-established severity illness and mortality risk scores were assessed to identify risk factors associated with NICU care^18–22^. Although these factors were mostly validated among VLBW infants for neonatal mortality, we considered them to be relevant to an infant’s health status beyond VLBW and associated with NICU admission^6,22^. Among known risk factors, gestational age using the obstetric estimate of gestation at delivery (OE), small for gestational age (SGA), large for gestational age (LGA), 5-min Apgar, plurality, cesarean delivery, and sex were associated with NICU admission and included in the risk adjustment^6,18–23^. Details of the modeling strategy are presented in the supplemental materials.

### Statistical analysis

Analyses were conducted overall and stratified by birth weight group as VLBW (< 1500 g), moderately low birth weight (MLBW, 1500–2499 g) and normal to high birth weight (NHBW, ≥ 2500 g). Univariable analyses were conducted to assess association between each of risk factors -gestational age, SGA, LGA, 5-min Apgar, plurality, cesarean delivery, and sex- and NICU admission. Multivariable logistic regression models were specified with NICU admission as the dependent variable and race/ethnicity as a primary independent variable while adjusting for birth year and risk factors that were statistically significant in univariable analysis. To assess differential temporal trends for NICU admission across race/ethnicity, we included interaction terms between birth year and race/ethnicity in the models. The model-adjusted NICU admission rates were estimated with predicted probabilities using Stata command *margins* based on marginal standardization method and adjusted rate ratios (ARRs) for black and Hispanic infants compared with white infants were estimated using Stata command *nlcom*^24^. Stata command *margins* produces the adjusted predictions that are expected values of a dependent variable computed from the results of a regression where all other covariates are held at means. The *nlcom* takes nonlinear transformations of a parameter estimate from a fitted model to estimate the risk ratio and construct the confidence interval using the delta approximation method, an approximation appropriate in large samples.

We assessed the representativeness of our study cohort by comparing birth cohorts recorded with the 2003 revision to total U.S. birth cohorts for 2008–2015 before the 2003 revision had been implemented in all U.S. states and the District of Columbia. We conducted sensitivity analyses for temporal trends limiting analysis to births that occurred in the 27 U.S. states where the 2003 revision had been used throughout the entire study period (2008–2018). All statistical analyses were performed using Stata, version 16.0 (StataCorp LLC., College Station, TX, USA).

## Results

**Figure 1:**
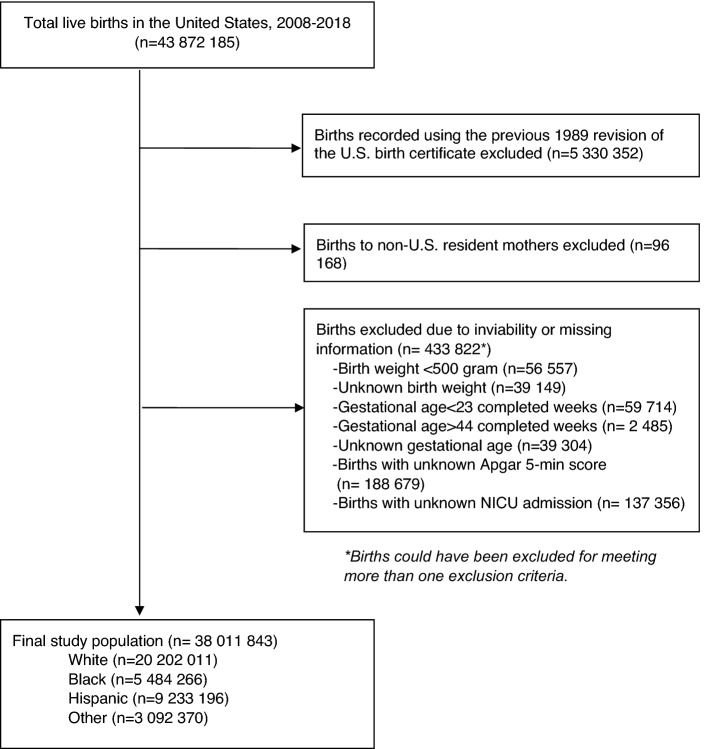
Cohort derivation.

From 2008 to 2018, there were 43,872,185 live births. Of these, 38,011,843 births were included in the study sample (Fig. 1). In the study sample, 53.1% were white, 14.4% were black, and 24.3% were Hispanic (Table 1). Black and Hispanic mothers were twice more likely than white mothers to be adolescent, unmarried, and receive Medicaid and WIC (the special supplemental nutrition program for women, infants, and children). Black and Hispanic mothers had lower education levels and lived in large central metro areas. The percentage of cesarean delivery was 32.4% and slightly higher among black infants than among white and Hispanic infants (Table 1). Percentages of VLBW and LBW were two times higher among black infants compared with white and Hispanic infants.

**Table 1:** Maternal and neonatal characteristics of live births: United States, 2008–2018.

Mother's race/ethnicity	Births, No. (%)				
	All	White	Black	Hispanic	Other
No. of births	38,011,843	20,202,011	5,484,266	9,233,196	3,092,370
% of births	100.0	53.1	14.4	24.3	8.1
**Maternal characteristics, %**					
Age category, year					
< 20	7.1	5.2	10.9	10.6	3.0
20–24	22.2	19.8	29.9	26.0	12.2
25–29	28.7	30.0	27.3	27.5	26.6
30–34	26.2	28.9	19.5	21.6	34.3
35–39	12.8	13.2	9.9	11.5	19.2
40–54	3.0	2.9	2.6	2.9	4.8
Payment source^a^					
Medicaid	43.0	31.2	65.1	59.7	31.5
Private Insurance	47.5	61.0	26.8	26.4	57.8
Self-Pay	4.1	2.9	2.9	7.3	5.0
Other	4.4	4.0	4.1	5.5	4.7
Unknown	1.1	1.0	1.0	1.2	1.1
WIC received^a^	42.4	29.0	61.5	64.8	29.9
Unmarried^b^	40.4	29.2	71.0	52.6	22.8
Educational attainment					
Less than high school	16.1	8.5	17.3	34.1	9.8
High school	25.1	21.9	33.4	30.4	16.3
Some college	20.5	21.1	26.4	18.0	13.4
Associate or Bachelor's degree	26.5	34.1	16.8	13.1	33.6
Master's or higher degree	10.6	13.9	5.1	3.1	20.9
Unknown	1.2	0.5	0.9	1.3	6.0
Urban–rural classification^c^					
Large central metro	38.4	28.4	47.2	49.5	55.5
Large fringe metro	19.5	21.0	20.5	16.4	17.8
Medium metro	22.4	24.3	19.1	22.6	14.7
Small metro	9.9	12.9	7.6	6.2	5.6
Micropolitan	7.7	10.5	4.6	4.4	4.7
Noncore	2.1	3.0	1.0	0.9	1.8
**Neonatal characteristics, %**					
Female	48.8	48.7	49.2	49.0	48.5
Multiple gestations	3.4	3.7	3.9	2.4	3.2
Cesarean delivery	32.4	31.6	35.7	32.0	32.7
Birthweight category, g					
< 1500	1.2	1.0	2.5	1.1	1.1
1500–2499	6.7	5.9	10.5	5.9	7.2
2500–3999	84.3	83.6	82.7	85.9	86.3
≥ 4000	7.8	9.5	4.4	7.1	5.5
Gestational age, week					
< 32	1.4	1.1	2.6	1.3	1.2
32–36	8.2	7.8	10.5	7.8	7.8
37–38	26.3	24.7	28.6	27.9	28.0
39–40	57.5	59.0	52.7	57.1	57.1
≥ 41	6.7	7.4	5.6	6.0	5.9
5-Minute Apgar score					
< 7	1.8	1.8	3.0	1.3	1.4
7–8	12.6	13.6	14.2	10.1	10.8
9–10	85.5	84.5	82.8	88.6	87.8

^a^Information was restricted to births since 2009 when they started to be collected.

^b^Births occurring in or to residents of California in 2017–2018 were excluded due to state statutory restrictions.

^c^The National Center for Health Statistics’ (NCHS) Urban–Rural Classification Scheme for Counties was used.

### Trends for crude NICU admission rates by race/ethnicity

**Figure 2:**
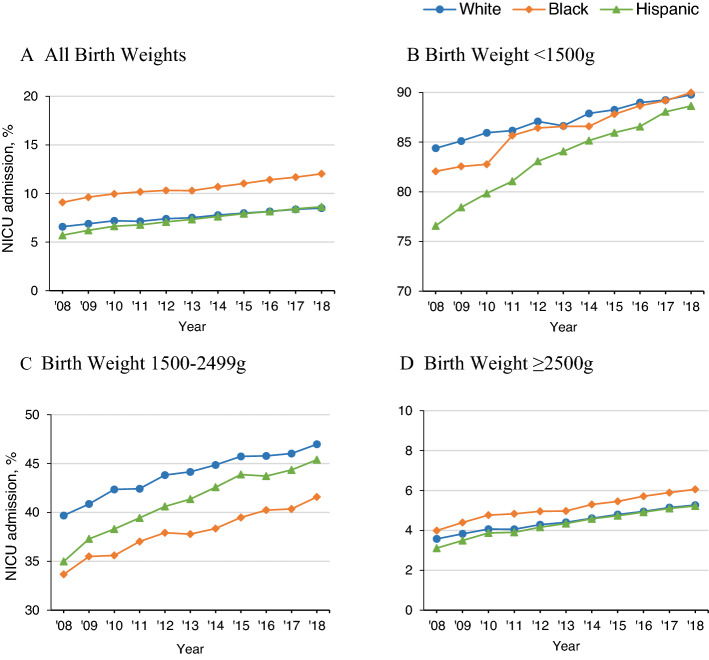
Temporal trends for crude NICU admission rates by race/ethnicity for 2008–2018 all births and subgroups.

Overall crude NICU admission rates increased from 6.62% (95% CI 6.59–6.65) in 2008 to 9.07% (95% CI 9.04–9.10) in 2018, a 37% growth. Increases were observed regardless of race/ethnicity: 6.58% (95% CI 6.54–6.62) to 8.50% (95% CI 8.46–8.53) among white infants, 9.09% (95% CI 8.99–9.18) to 12.03% (95% CI 11.94–12.11) among black infants, and 5.70% (95% CI 5.65–5.75) to 8.63% (95% CI 8.57–8.69) among Hispanic infants (Fig. 2; Supplemental Tables 1–4). NICU admission rates were the highest among black infants across all years. Among Hispanic infants, the NICU admission rate was the lowest in 2008, but it increased the most with the largest percent change (51.4%) compared with white (29.1%) and black (32.4%) infants.

**Figure 3:**
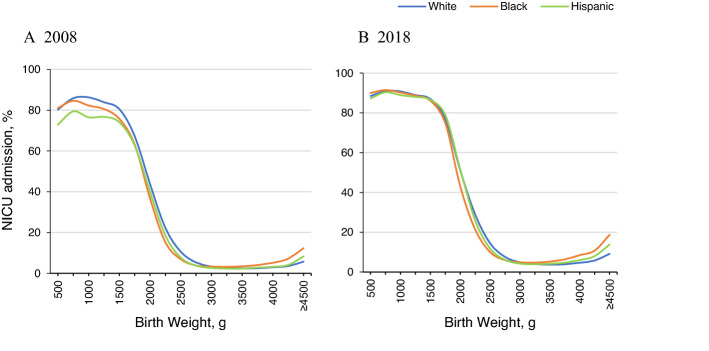
NICU admission across birth weight by race/ethnicity, 2008 vs 2018.

In the birth weight-stratified analysis, white infants had higher NICU admission rates in the VLBW and MLBW groups, whereas black infants had higher NICU admission rates in the NHBW group (Fig. 2). The differences in NICU admission rates among the highest risk group (VLBW) were prominent between white and Hispanic infants in 2008, but greatly decreased. In the MBLW group, the differences in NICU admission rates between white and black infants remained persistent over the study period. Figure 3 shows how racial/ethnic differences of NICU admission rates across birth weights changed between 2008 and 2018. Similar trends emerged in the gestational age-stratified analysis. White infants had higher NICU admission rates in lower gestational age groups (≤ 36 weeks), whereas black infants had higher NICU admission rates in higher gestational age groups (> 36 weeks) (Supplemental Fig. 1).

### Trends for NICU admission rate ratios by race/ethnicity

Table 2 shows trends for rate ratios of NICU admission between black and white infants and between Hispanic and white infants overall and by birth weight groups. Black infants had an approximately 40% higher rate of NICU admission than white infants over the study period, whereas Hispanic infants had a 12% lower NICU admission rate than white infants in 2008 but reached the same rate by 2015. Overall risk-adjusted rate ratios remained close to 1 for both black and Hispanic infants. However, birth weight stratified analyses showed different trends. Between black and white infants, adjusted rate ratios remained close to 1 in the VLBW group, slightly lower in the MLBW group but higher in the NHBW group. Between Hispanic and white infants, adjusted rate ratios were lower than 1 but increased over the years in the VLBW and MLBW groups but continued to be higher than 1 in the NHBW group.

**Table 2:** Trends for crude and adjusted rate ratios for NICU admission among black and hispanic infants compared with white infants, 2008–2018.

	2008	2009	2010	2011	2012	2013	2014	2015	2016	2017	2018
**NICU, all**	6.62	7.02	7.43	7.48	7.74	7.90	8.17	8.44	8.66	8.90	9.07
Black RR	1.38 (1.36–1.40)	1.40 (1.38–1.41)	1.38 (1.37–1.40)	1.42 (1.41–1.44)	1.39 (1.38–1.41)	1.37 (1.36–1.38)	1.37 (1.36–1.39)	1.38 (1.37–1.39)	1.40 (1.39–1.41)	1.40 (1.38–1.41)	1.42 (1.40–1.43)
Hispanic RR	0.88 (0.86–0.88)	0.91 (0.89–0.91)	0.93 (0.91–0.93)	0.96 (0.94–0.96)	0.96 (0.95–0.96)	0.99 (0.97–0.99)	0.99 (0.97–0.99)	1.00 (0.98–1.00)	1.01 (0.99–1.01)	1.01 (1.00–1.01)	1.02 (1.01–1.02)
Black ARR	1.03 (1.03–1.04)	1.03 (1.03–1.04)	1.04 (1.03–1.04)	1.04 (1.03–1.04)	1.04 (1.04–1.04)	1.04 (1.04–1.04)	1.04 (1.04–1.05)	1.05 (1.04–1.05)	1.05 (1.04–1.05)	1.05 (1.05–1.05)	1.05 (1.05–1.06)
Hispanic ARR	0.98 (0.98–0.99)	0.99 (0.98–0.99)	0.99 (0.99–1.00)	1.00 (1.00–1.00)	1.01 (1.01–1.01)	1.02 (1.01–1.02)	1.02 (1.02–1.02)	1.03 (1.03–1.03)	1.04 (1.03–1.04)	1.04 (1.04–1.05)	1.05 (1.05–1.05)
**NICU, VLBW**	81.44	82.53	83.47	84.59	85.75	85.97	86.60	87.54	88.33	88.86	89.42
Black RR	0.97 (0.96–0.98)	0.97 (0.96–0.98)	0.96 (0.95–0.97)	0.99 (0.98–1.00)	0.99 (0.98–1.00)	1.00 (0.99–1.01)	0.99 (0.98–0.99)	0.99 (0.99–1.00)	1.00 (0.99–1.00)	1.00 (0.99–1.01)	1.00 (0.99–1.01)
Hispanic RR	0.91 (0.89–0.92)	0.92 (0.91–0.93)	0.93 (0.92–0.94)	0.94 (0.93–0.95)	0.95 (0.94–0.96)	0.97 (0.96–0.98)	0.97 (0.96–0.98)	0.97 (0.96–0.98)	0.97 (0.96–0.98)	0.99 (0.98–1.00)	0.99 (0.98–1.00)
Black ARR	0.98 (0.97–0.98)	0.98 (0.98–0.99)	0.98 (0.98–0.99)	0.99 (0.98–0.99)	0.99 (0.99–0.99)	0.99 (0.99–1.00)	0.99 (0.99–1.00)	1.00 (0.99–1.00)	1.00 (1.00–1.00)	1.00 (1.00–1.00)	1.00 (1.00–1.01)
Hispanic ARR	0.92 (0.91–0.93)	0.93 (0.92–0.94)	0.94 (0.93–0.94)	0.95 (0.94–0.95)	0.96 (0.95–0.96)	0.96 (0.96–0.97)	0.97 (0.97–0.97)	0.98 (0.97–0.98)	0.98 (0.98–0.99)	0.99 (0.98–0.99)	0.99 (0.99–1.00)
**NICU, MLBW**	36.74	38.29	39.39	40.04	41.14	41.49	42.29	43.26	43.49	43.76	44.67
Black RR	0.85 (0.83–0.86)	0.87 (0.85–0.88)	0.84 (0.83–0.85)	0.87 (0.86–0.88)	0.87 (0.85–0.88)	0.86 (0.84–0.87)	0.85 (0.84–0.87)	0.86 (0.85–0.87)	0.88 (0.87–0.89)	0.88 (0.87–0.89)	0.89 (0.88–0.89)
Hispanic RR	0.88 (0.87–0.89)	0.91 (0.90–0.93)	0.90 (0.89–0.92)	0.93 (0.92–0.94)	0.93 (0.91–0.94)	0.94 (0.93–0.95)	0.95 (0.94–0.96)	0.96 (0.95–0.97)	0.95 (0.94–0.97)	0.96 (0.95–0.97)	0.97 (0.96–0.98)
Black ARR	0.96 (0.95–0.97)	0.96 (0.96–0.97)	0.96 (0.96–0.97)	0.97 (0.96–0.97)	0.97 (0.96–0.97)	0.97 (0.97–0.97)	0.97 (0.97–0.97)	0.97 (0.97–0.98)	0.98 (0.97–0.98)	0.98 (0.97–0.98)	0.98 (0.97–0.98)
Hispanic ARR	0.95 (0.94–0.96)	0.96 (0.95–0.96)	0.96 (0.96–0.97)	0.97 (0.96–0.97)	0.97 (0.97–0.98)	0.98 (0.98–0.98)	0.98 (0.98–0.99)	0.99 (0.99–0.99)	0.99 (0.99–1.00)	1.00 (0.99–1.00)	1.00 (1.00–1.01)
**NICU, NHBW**	3.47	3.79	4.10	4.12	4.35	4.47	4.70	4.88	5.05	5.23	5.36
Black RR	1.12 (1.09–1.14)	1.15 (1.13–1.17)	1.17 (1.15–1.19)	1.19 (1.17–1.21)	1.16 (1.14–1.17)	1.13 (1.11–1.15)	1.15 (1.13–1.17)	1.14 (1.12–1.15)	1.15 (1.14–1.17)	1.15 (1.13–1.16)	1.15 (1.14–1.16)
Hispanic RR	0.87 (0.86–0.88)	0.91 (0.90–0.93)	0.95 (0.94–0.97)	0.96 (0.95–0.98)	0.97 (0.96–0.98)	0.99 (0.97–1.00)	0.99 (0.98–1.01)	0.99 (0.98–1.00)	0.99 (0.98–1.00)	0.99 (0.98–1.00)	0.99 (0.98–1.00)
Black ARR	1.08 (1.07–1.09)	1.08 (1.07–1.09)	1.08 (1.07–1.08)	1.08 (1.07–1.08)	1.07 (1.07–1.08)	1.07 (1.07–1.08)	1.07 (1.07–1.08)	1.07 (1.07–1.08)	1.07 (1.07–1.08)	1.07 (1.06–1.08)	1.07 (1.06–1.08)
Hispanic ARR	1.02 (1.01–1.03)	1.03 (1.02–1.03)	1.03 (1.03–1.04)	1.04 (1.04–1.05)	1.05 (1.04–1.05)	1.05 (1.05–1.06)	1.06 (1.06–1.06)	1.07 (1.06–1.07)	1.07 (1.07–1.08)	1.08 (1.08–1.09)	1.09 (1.08–1.09)

*NICU* neonatal intensive care unit, *RR* rate ratio, *ARR* adjusted rate ratio, *VLBW* very low birth weight (< 1500 g), *MLBW* moderately low birth weight (1500–2499 g), *NHBW* normal to high birth weight (≥ 2500 g).

### Sensitivity analysis

Maternal and neonatal characteristics were similar between births recorded with the revised version of birth certificate and total births, suggesting that our study population represented national trends in NICU admission (Supplemental Table 5). When we limited our analysis to births in the U.S. states where the 2003 revision had been used throughout the entire study period, we found that trends in NICU admission overall and by race/ethnicity were consistent (Supplemental Table 6).

## Discussion

Overall NICU admission rates increased by 37% from 2008 to 2018, and the increasing trends were observed among all racial and ethnic groups. The absolute and percent increases were the smallest among white infants. NICU admission rates among black infants remained highest and increased to 12% in 2018. Hispanic infants had lowest NICU admission rates in 2008 but reached rates similar to those of white infants in 2018.

Most differences in overall NICU admission rates by race/ethnicity disappeared after risk adjustment. This could indicate that crude racial/ethnic rate differences were justified by different risks or needs. These average findings, however, obscure important differences revealed in stratified analyses. In the VLBW group, compared to white infants, black infants had similar NICU admission rates while Hispanic infants had much lower NICU admission rates that were catching up by 2018. Only 77% of Hispanic VLBW infants were admitted to a NICU in 2008 while 84% of white and 82% of black VLBW infants were admitted to a NICU. But 90% of white and black VLBW infants and 89% of Hispanic VLBW infants were admitted to a NICU in 2018.This may reflect improved access to timely appropriate NICU care among high-risk infants through increasing health care coverage coupled with growing NICU supply^25,26^. Higher rates of NICU admissions with little racial/ethnic differences among high risk infants, especially VLBW infants who are recommended to be admitted to a NICU according to the AAP guideline, are encouraging trends in perinatal care. In the MLBW group, NICU admission rates in black infants compared to white infants remained lowest, while lower rates in Hispanic infants became similar to those of white infants over the study period. This seems associated with lower other health risk such as lower multiple births (19% vs 28%) among black infants compared to white infants in the MLBW group but may also imply unmet need for black MLBW infants.

In contrast, black infants maintained higher risk-adjusted NICU admission rates in the NHBW group. Black infants in this group had about 15% higher NICU admission rates compared to white infants even though differences became half (7%) after risk adjustment. Hispanic infants in this group had lower NICU admission rates compared to white infants in 2008 but similar rates in 2018. Their risk adjusted rates were similar to those of white infants in 2008 but became higher in 2018. Traditional risk factors might have under-adjusted health risk in the NHBW group even though we included large for gestational age. However, opposite directions of risk adjustment in black (crude RR 1.15 vs adjusted RR 1.07 in 2018) and Hispanic infants (crude RR 0.99 vs adjusted 1.07 in 2018) led us to look for other explanation. The higher use of NICUs in this low risk group may also indicate overutilization of NICUs^27^. The growth in NICU bed supply has outpaced measured need^25^, and the greater availability of NICU beds is known to be associated with greater utilization^9,28^. Possible overuse of NICU care by race and ethnicity is an important area of future research. Freeman demonstrated that available NICU beds increased additional NICU utilization among those less ill or in the range of birth weights in which admission decisions are likely to be more discretionary^28^. Shulman et al. found that among infants born at GA of 34 weeks or more, inborn admission rates for specific GA strata correlated strongly with overall inborn admission rates and did not significantly correlate with percentage of admissions with high illness acuity^7^. Similarly, Ziegler et al. found significant between-hospital variation in NICU admission rates that cannot be explained by infant health condition among infants born 35 to 42 weeks' gestation^8^. Harrison et al. found that non-VLBW infants were more likely to be admitted to a NICU in regions with the highest NICU bed supply, indicating possible overuse^9^. In our study population, almost 50% of the black and Hispanic mothers compared with 28% of white mothers resided in a large central metro area where they were likely to be close to large hospitals with NICU beds^26,29,30^. Increased capacity, payments that reward NICU care, perhaps disproportionately to its value in lower risk newborns, and weak state regulation may cause potential overuse of NICU among NHBW infants^10,31,32^. The U.S. has significantly greater neonatal clinicians and NICU beds per capita than other developed countries with provision of neonatal intensive care extended beyond regional or academic centers^5,33^. Yet, there lacks of clear criteria for designating levels of risk-appropriate neonatal care and capability across states^31^.

### Strengths and limitations

This study is one of few that compares NICU utilization by race/ethnicity and evaluates the effect of race/ethnicity on NICU admission rate as a primary interest across all birth weights^22,26,29^. The literature is rich in evaluating the quality of care across providers or hospital characteristics, but it is often limited to VLBW or VPT infants. When race/ethnicity is included, it is usually as a covariate in statistical modeling rather than as a primary study exposure^34,35^. Understanding racial/ethnic differences in NICU admission is particularly relevant given that racial/ethnic differences in birth outcomes are persistent^36^.

This study has some limitations. First, even though the birth certificate form defines NICU admissions, differences in coding may have occurred across states and hospitals. A 2012 policy statement by the AAP defines a NICU as a level III and IV facilities, where ongoing assisted ventilation for 24 h or more is available^2^, but there is a wide variation among states in the definition and criteria of a NICU and accuracy of coding may improve over the years^37^. However, increasing NICU admission trends were observed also in hospital discharge data^28^ and validation studies on the accuracy of birth certificate data report a good agreement on NICU admission between birth certificates compared to hospital medical records^38^. A validation study assessing the quality of medical and health data from the birth certificate from two states found levels of agreement or sensitivity for most checkbox items were substantial or moderate and the sensitivity for NICU admission from a state using the stratified systematic sampling methodology was over 95%^38^. Second, our study may have underestimated NICU admissions. Since birth certificates are required to be filed within 5 days of the date of birth by the birth hospital, late NICU admissions in a prolonged hospitalization occurring after the filing or NICU admissions occurring at the transferred hospital may not be included^39,40^. Also, the infants staying at the NICU for observation without being admitted to NICU are not counted as NICU admission according to CDC’s guideline^17^. A recent study examining the concordance of hospital-level NICU admission rates between birth certificate data and hospital reported NICU data by all hospitals with an accredited NICU in California finds that birth certificates tends to underreport NICU admissions but the study suggests that good agreement between hospital ranks that are based on NICU admission rates from birth certificates and the gold standard reflects the relative performance of a hospital on NICU admissions^40^. In an additional sensitivity analysis assuming transferred infants were all admitted to NICU, we found NICU admission rates in 2018 changed from 8.5 to 8.9% in white infants, from 12.0 to 12.3% in black infants, and from 8.6 to 8.9% in Hispanic infants. When limited to VLBW infants to be considered NICU admitted with transfer, we found a little changed: 89.8–91.5% in white infants, 90.0–91.7% in black infants and 88.6–90.3%. Given that our study focus was to measure relative use of NICU by racial/ethnic minority compared to white infants and changes in temporal trend in a population level rather than the exact rate of NICU admissions, our findings should provide reasonable information to assess racial/ethnic differences in NICU admissions and temporal trends.

## Conclusions

From 2008 to 2018, there was little difference in overall risk-adjusted NICU admission rates by race/ethnicity. However, birth weight-stratified analysis revealed that racial/ethnic differences diminished in the VLBW and MLBW groups while risk-adjusted NICU admission rates remained higher among black and Hispanic infants in the NHBW group. The increasing trend in NICU admission rates among the NHBW group appear to continue and further study is needed to identify the reasons for this trend and prevent possible overuse of NICU care.

## Supplementary Information

Supplementary Information.
