# Supplementary material for: Trends in neonatal intensive care unit admissions by race/ethnicity in the United States, 2008–2018
Source: Sci Rep. 2021 Dec 10;11:23795. doi: 10.1038/s41598-021-03183-1 (PMC8664880; doi:10.1038/s41598-021-03183-1)
Supplement: Supplementary file 1 — Supplementary Information. [file 41598_2021_3183_MOESM1_ESM.docx]

**Supplemental Digital Content**

Modeling Strategy for Risk Adjustment

Supplemental Table 1. Temporal Trends for Crude NICU Admission Rates for 2008-2018 by Race/Ethnicity, All Birth Weights

Supplemental Table 2. Temporal Trends for Crude NICU Admission Rates for 2008-2018 by Race/Ethnicity, Birth Weight <1500g

Supplemental Table 3. Temporal Trends for Crude NICU Admission Rates for 2008-2018 by Race/Ethnicity, Birth Weight 1500-2499g

Supplemental Table 4. Temporal Trends for Crude NICU Admission Rates for 2008-2018 by Race/Ethnicity, Birth Weight ≥2500g

Supplemental Fig 1. Temporal Trends for NICU Admission by Gestational Age and Race/Ethnicity for 2008-2018

Supplemental Table 5. Comparison of the Study Population and Total U.S. Birth Cohorts for 2008-2015

Supplemental Table 6. Temporal Trends for Crude NICU Admission Rates for 2008-2018 by Race/Ethnicity, All Birth Weights Limited to 27 States

Reference

**Modeling Strategy for Risk Adjustment**

We selected potential risk factors mainly from four well-known risk adjustment scores that have been widely used in the United States and the United Kingdom and included factors beyond physiologic variables, which were not available on the birth certificate.^1-5^ When the updated or improved versions of risk adjustment score models were available, most updated ones were used (SNAP-PE II and CRIB II).

**Comparison of Data Components in Mortality Risk Adjustment Scores for Neonatal Intensive Care Unit Settings**

|  | **NICU Mortality Risk Adjustment Scores** | | | |
| --- | --- | --- | --- | --- |
|  | SNAP-PE II | CRIB II | VON-RA | NICHD 2008 |
| Population |  |  |  |  |
| All NICU patients | + | + | + |  |
| <1500 g birth weight |  |  |  |  |
| 22 to 25 wk gestation |  |  |  | + |
| Excludes lethal anomalies |  |  |  | + |
| <31 wk gestation |  |  |  |  |
| <32 wk gestation |  | + |  |  |
| Birth characteristics |  |  |  |  |
| Birth weight | + | + |  | + |
| Small for gestational age | + |  | + |  |
| Apgar score | + |  | + |  |
| Gestational age |  | + | + | + |
| Birth defects |  |  | + |  |
| Gender |  | + | + | + |
| Plurality |  |  | + | + |
| Antenatal steroids |  |  |  | + |
| Transfer |  |  | + |  |
| Mode of delivery |  |  | + |  |
| Clinical characteristics | + | + |  |  |

We excluded birth defect and transfer status because of their infrequency or unreliability. The definition of birth defect was inconsistent, and its infrequency was not expected to add discrimination because almost all the infants were without a birth defect. Transfer was merely a proxy for NICU admission and was also not expected to add discrimination because almost all infants were inborn. Gestational age was in completed weeks, and small for gestational age (SGA, Yes or No) and large for gestational age (LGA, Yes or NO) were defined as below the 10th percentile and above the 97 percentiles for birth weight respectively, given the infant’s gestational age, race/ethnicity and gender based on the United States 2008-2018 natality data.

Univariable logistic regressions for each of the candidate risk factors versus NICU admission were conducted, and area under receiver operator characteristic (ROC) curve was assessed. Within each variable, adjacent risk categories were consolidated if their area under ROCs did not differ significantly.

**Univariable Logistic Regression for Potential Risk Factors**

|  | **Coeff.** | **Std. Err.** | **z** | **p-value** | **95% CI** | |
| --- | --- | --- | --- | --- | --- | --- |
| **Gestational Age** |  |  |  | <0.001^a^ |  |  |
| <32 | 5.194 | 0.004 | 1266.36 | <0.001 | 5.186 | 5.202 |
| 32 | 4.986 | 0.006 | 789.24 | <0.001 | 4.974 | 4.999 |
| 33 | 4.826 | 0.005 | 949.18 | <0.001 | 4.816 | 4.836 |
| 34 | 4.365 | 0.003 | 1273.03 | <0.001 | 4.358 | 4.371 |
| 35 | 2.989 | 0.003 | 1112.58 | <0.001 | 2.984 | 2.994 |
| 36 | 1.896 | 0.002 | 766.53 | <0.001 | 1.892 | 1.901 |
| 37 | 0.964 | 0.002 | 410.37 | <0.001 | 0.959 | 0.968 |
| 38 | 0.270 | 0.002 | 117.14 | <0.001 | 0.266 | 0.275 |
| 39-40 | Reference |  |  |  |  |  |
| >=41 | 0.299 | 0.003 | 89.16 | <0.001 | 0.292 | 0.305 |
| **5-min Apgar Score** | |  |  | <0.001^a^ |  |  |
| <7 | 2.964 | 0.003 | 1176.95 | <0.001 | 2.959 | 2.969 |
| 7-8 | 1.538 | 0.001 | 1116.5 | <0.001 | 1.535 | 1.541 |
| 9-10 | Reference |  |  |  |  |  |
| **Cesarean Delivery** | 1.038 | 0.001 | 859.4 | <0.001 | 1.036 | 1.041 |
| **Plurality** | 2.126 | 0.002 | 1103.62 | <0.001 | 2.122 | 2.130 |
| **SGA** | 0.497 | 0.002 | 289.47 | <0.001 | 0.494 | 0.501 |
| **Female Sex** | -0.170 | 0.001 | -142 | <0.001 | -0.173 | -0.168 |
| **LGA** | 0.284 | 0.003 | 90.02 | <0.001 | 0.278 | 0.290 |

^a^Factor p-value for overall significance.

**Area under the ROC from Univariable Regressions**

|  | Area Under ROC curve | Std. Err. | 95% CI | |
| --- | --- | --- | --- | --- |
| Gestational Age | 0.7932 | 0.0002 | 0.79288 | 0.79352 |
| 5-min Apgar Score | 0.6659 | 0.0001 | 0.66564 | 0.66622 |
| Cesarean Delivery | 0.6242 | 0.0001 | 0.62387 | 0.62445 |
| Plurality | 0.5697 | 0.0001 | 0.56952 | 0.56993 |
| SGA | 0.5260 | 0.0001 | 0.5258 | 0.52621 |
| Female Sex | 0.5212 | 0.0001 | 0.52091 | 0.52149 |
| LGA | 0.5046 | 0.0001 | 0.50446 | 0.50469 |

We built multivariable models by starting with gestational age and adding a risk factor in the model one by one based on the area under ROC from univariable regressions. We compared models for predictive performance using the area under ROC. Our final model included gestational age, 5-min Apgar score, cesarean delivery, plurality, SGA, female sex and LGA. The area under ROC was 0.8581, which was considered excellent discrimination.^6^

**Multivariable Logistic Regressions**

|  | **Model 1** | | **Model 2** | | **Model 3** | | **Model 4** | | **Model 5** | | **Model 6** | | **Model 7** | |
| --- | --- | --- | --- | --- | --- | --- | --- | --- | --- | --- | --- | --- | --- | --- |
| Gestational Age | Coef. | SE | Coef. | SE | Coef. | SE | Coef. | SE | Coef. | SE | Coef. | SE | Coef. | SE |
| <32 | 5.194 | 0.004 | 4.585 | 0.004 | 4.435 | 0.004 | 4.387 | 0.004 | 4.448 | 0.004 | 4.449 | 0.004 | 4.447 | 0.004 |
| 32 | 4.986 | 0.006 | 4.756 | 0.006 | 4.625 | 0.007 | 4.570 | 0.007 | 4.630 | 0.007 | 4.628 | 0.007 | 4.628 | 0.007 |
| 33 | 4.826 | 0.005 | 4.685 | 0.005 | 4.579 | 0.005 | 4.526 | 0.005 | 4.583 | 0.005 | 4.581 | 0.005 | 4.582 | 0.005 |
| 34 | 4.365 | 0.003 | 4.279 | 0.004 | 4.197 | 0.004 | 4.148 | 0.004 | 4.199 | 0.004 | 4.198 | 0.004 | 4.199 | 0.004 |
| 35 | 2.989 | 0.003 | 2.878 | 0.003 | 2.797 | 0.003 | 2.750 | 0.003 | 2.785 | 0.003 | 2.783 | 0.003 | 2.783 | 0.003 |
| 36 | 1.896 | 0.002 | 1.795 | 0.003 | 1.722 | 0.003 | 1.684 | 0.003 | 1.706 | 0.003 | 1.703 | 0.003 | 1.703 | 0.003 |
| 37 | 0.964 | 0.002 | 0.906 | 0.002 | 0.862 | 0.002 | 0.841 | 0.002 | 0.849 | 0.002 | 0.847 | 0.002 | 0.846 | 0.002 |
| 38 | 0.270 | 0.002 | 0.262 | 0.002 | 0.255 | 0.002 | 0.247 | 0.002 | 0.247 | 0.002 | 0.245 | 0.002 | 0.244 | 0.002 |
| 39-40 | reference | | reference | | reference | | reference | | reference | | reference | | reference | |
| ≥41 | 0.299 | 0.003 | 0.239 | 0.003 | 0.271 | 0.003 | 0.270 | 0.003 | 0.270 | 0.003 | 0.269 | 0.003 | 0.269 | 0.003 |
| 5-min Apgar |  |  |  |  |  |  |  |  |  |  |  |  |  |  |
| <7 |  |  | 2.406 | 0.003 | 2.358 | 0.003 | 2.363 | 0.003 | 2.341 | 0.003 | 2.336 | 0.003 | 2.332 | 0.003 |
| 7-8 |  |  | 1.143 | 0.002 | 1.120 | 0.002 | 1.121 | 0.002 | 1.117 | 0.002 | 1.117 | 0.002 | 1.115 | 0.002 |
| 9-10 |  |  | reference | | reference | | reference | | reference | | reference | | reference | |
| C-section |  |  |  |  | 0.634 | 0.001 | 0.613 | 0.002 | 0.605 | 0.002 | 0.604 | 0.002 | 0.595 | 0.002 |
| Plurality |  |  |  |  |  |  | 0.216 | 0.003 | 0.143 | 0.003 | 0.149 | 0.003 | 0.164 | 0.003 |
| SGA |  |  |  |  |  |  |  |  | 0.661 | 0.002 | 0.660 | 0.002 | 0.675 | 0.002 |
| Female Sex |  |  |  |  |  |  |  |  |  |  | -0.164 | 0.002 | -0.164 | 0.002 |
| LGA |  |  |  |  |  |  |  |  |  |  |  |  | 0.359 | 0.004 |
| **Area Under ROC** | 0.7932 | | 0.8415 | | 0.8528 | | 0.8529 | | 0.8558 | | 0.8571 | | 0.8581 | |
| (95% CI) | (0.7929-0.7935) | | (0.8412-0.8418) | | (0.8525-0.8531) | | (0.8527-0.8532) | | (0.8555-0.8560) | | (0.8569-0.8574) | | (0.8578-0.8583) | |

**Supplemental Table 1. Temporal Trends for Crude NICU Admission Rates for 2008-2018 by Race/Ethnicity, All Birth Weights**

|  | NICU admission, % (95% CI) | | | |
| --- | --- | --- | --- | --- |
| Year | All | White | Black | Hispanic |
| 2008 | 6.62 (6.59-6.65) | 6.58 (6.54-6.62) | 9.09 (8.99-9.18) | 5.70 (5.65-5.75) |
| 2009 | 7.02 (6.99-7.05) | 6.89 (6.84-6.93) | 9.62 (9.53-9.72) | 6.21 (6.15-6.26) |
| 2010 | 7.43 (7.40-7.45) | 7.19 (7.15-7.23) | 9.96 (9.87-10.05) | 6.64 (6.59-6.70) |
| 2011 | 7.48 (7.45-7.51) | 7.14 (7.10-7.18) | 10.17 (10.09-10.26) | 6.77 (6.72-6.83) |
| 2012 | 7.74 (7.71-7.77) | 7.40 (7.36-7.44) | 10.32 (10.23-10.40) | 7.07 (7.02-7.13) |
| 2013 | 7.90 (7.87-7.92) | 7.52 (7.48-7.55) | 10.29 (10.21-10.37) | 7.34 (7.28-7.40) |
| 2014 | 8.17 (8.14-8.20) | 7.78 (7.74-7.81) | 10.68 (10.60-10.76) | 7.64 (7.58-7.69) |
| 2015 | 8.44 (8.41-8.47) | 7.99 (7.95-8.02) | 11.03 (10.95-11.11) | 7.93 (7.87-7.98) |
| 2016 | 8.66 (8.63-8.68) | 8.16 (8.12-8.20) | 11.42 (11.33-11.50) | 8.15 (8.09-8.20) |
| 2017 | 8.90 (8.87-8.93) | 8.37 (8.33-8.41) | 11.68 (11.60-11.76) | 8.42 (8.36-8.48) |
| 2018 | 9.07 (9.04-9.10) | 8.50 (8.46-8.53) | 12.03 (11.94-12.11) | 8.63 (8.57-8.69) |

**Supplemental Table 2. Temporal Trends for Crude NICU Admission Rates for 2008-2018 by Race/Ethnicity, Birth Weight <1500g**

|  | NICU Admission, % (95% CI) | | | |
| --- | --- | --- | --- | --- |
| Year | All | White | Black | Hispanic |
| 2008 | 81.44 (81.01-81.86) | 84.39 (83.78-84.98) | 82.05 (81.23-82.86) | 76.58 (75.63-77.50) |
| 2009 | 82.53 (82.12-82.93) | 85.11 (84.52-85.69) | 82.55 (81.75-83.33) | 78.44 (77.51-79.34) |
| 2010 | 83.47 (83.09-83.84) | 85.94 (85.40-86.47) | 82.76 (82.03-83.48) | 79.84 (78.96-80.70) |
| 2011 | 84.59 (84.24-84.94) | 86.16 (85.65-86.66) | 85.66 (85.02-86.28) | 81.06 (80.20-81.90) |
| 2012 | 85.75 (85.41-86.08) | 87.07 (86.58-87.56) | 86.42 (85.81-87.03) | 83.07 (82.26-83.86) |
| 2013 | 85.97 (85.64-86.30) | 86.64 (86.14-87.12) | 86.58 (85.98-87.17) | 84.06 (83.27-84.84) |
| 2014 | 86.60 (86.28-86.91) | 87.89 (87.43-88.33) | 86.58 (85.99-87.15) | 85.15 (84.40-85.87) |
| 2015 | 87.54 (87.24-87.84) | 88.25 (87.80-88.69) | 87.81 (87.25-88.35) | 85.95 (85.23-86.64) |
| 2016 | 88.33 (88.04-88.62) | 88.98 (88.53-89.41) | 88.65 (88.12-89.17) | 86.56 (85.87-87.23) |
| 2017 | 88.86 (88.57-89.14) | 89.23 (88.78-89.67) | 89.17 (88.65-89.68) | 88.05 (87.39-88.69) |
| 2018 | 89.42 (89.13-89.70) | 89.77 (89.32-90.21) | 89.98 (89.47-90.47) | 88.64 (87.98-89.27) |

**Supplemental Table 3. Temporal Trends for Crude NICU Admission Rates for 2008-2018 by Race/Ethnicity, Birth Weight 1500-2499g**

|  | NICU Admission, % (95% CI) | | | |
| --- | --- | --- | --- | --- |
| Year | All | White | Black | Hispanic |
| 2008 | 36.74 (36.52-36.97) | 39.68 (39.35-40.01) | 33.66 (33.17-34.14) | 34.98 (34.54-35.42) |
| 2009 | 38.29 (38.07-38.51) | 40.86 (40.53-41.19) | 35.49 (35.01-35.98) | 37.29 (36.84-37.74) |
| 2010 | 39.39 (39.18-39.61) | 42.35 (42.04-42.66) | 35.60 (35.15-36.05) | 38.30 (37.86-38.75) |
| 2011 | 40.04 (39.83-40.24) | 42.42 (42.13-42.72) | 37.02 (36.60-37.45) | 39.44 (39.00-39.88) |
| 2012 | 41.14 (40.93-41.34) | 43.82 (43.53-44.12) | 37.91 (37.49-38.34) | 40.61 (40.16-41.06) |
| 2013 | 41.49 (41.29-41.69) | 44.15 (43.85-44.44) | 37.78 (37.36-38.19) | 41.36 (40.92-41.81) |
| 2014 | 42.29 (42.10-42.48) | 44.86 (44.58-45.14) | 38.35 (37.95-38.75) | 42.58 (42.15-43.01) |
| 2015 | 43.26 (43.07-43.46) | 45.73 (45.45-46.01) | 39.48 (39.09-39.87) | 43.88 (43.46-44.30) |
| 2016 | 43.49 (43.30-43.68) | 45.78 (45.50-46.06) | 40.23 (39.85-40.62) | 43.72 (43.30-44.13) |
| 2017 | 43.76 (43.57-43.95) | 46.02 (45.74-46.30) | 40.35 (39.97-40.73) | 44.35 (43.93-44.77) |
| 2018 | 44.67 (44.48-44.86) | 46.97 (46.69-47.26) | 41.58 (41.19-41.96) | 45.39 (44.97-45.81) |

**Supplemental Table 4. Temporal Trends for Crude NICU Admission Rates for 2008-2018 by Race/Ethnicity, Birth Weight ≥2500g**

|  | NICU Admission, % (95% CI) | | | |
| --- | --- | --- | --- | --- |
| Year | All | White | Black | Hispanic |
| 2008 | 3.47 (3.45-3.49) | 3.57 (3.54-3.61) | 3.99 (3.92-4.06) | 3.11 (3.07-3.15) |
| 2009 | 3.79 (3.76-3.81) | 3.83 (3.79-3.86) | 4.39 (4.32-4.47) | 3.49 (3.45-3.53) |
| 2010 | 4.10 (4.08-4.12) | 4.06 (4.03-4.09) | 4.76 (4.69-4.83) | 3.87 (3.83-3.91) |
| 2011 | 4.12 (4.10-4.14) | 4.05 (4.02-4.08) | 4.83 (4.76-4.90) | 3.90 (3.86-3.94) |
| 2012 | 4.35 (4.32-4.37) | 4.29 (4.26-4.32) | 4.96 (4.89-5.02) | 4.16 (4.11-4.20) |
| 2013 | 4.47 (4.45-4.49) | 4.40 (4.37-4.43) | 4.97 (4.91-5.03) | 4.34 (4.29-4.39) |
| 2014 | 4.70 (4.67-4.72) | 4.61 (4.58-4.64) | 5.30 (5.24-5.36) | 4.58 (4.53-4.62) |
| 2015 | 4.88 (4.86-4.90) | 4.80 (4.77-4.83) | 5.45 (5.39-5.52) | 4.73 (4.68-4.78) |
| 2016 | 5.05 (5.03-5.07) | 4.95 (4.92-4.98) | 5.71 (5.64-5.77) | 4.91 (4.87-4.96) |
| 2017 | 5.23 (5.21-5.26) | 5.14 (5.11-5.18) | 5.89 (5.83-5.96) | 5.10 (5.05-5.14) |
| 2018 | 5.36 (5.33-5.38) | 5.26 (5.23-5.30) | 6.06 (5.99-6.12) | 5.22 (5.18-5.27) |

**Supplemental Fig 1. Temporal Trends for NICU Admission by Gestational Age and Race/Ethnicity for 2008-2018**

A. Gestational Age <32 wk B. Gestational Age 32-36 wk


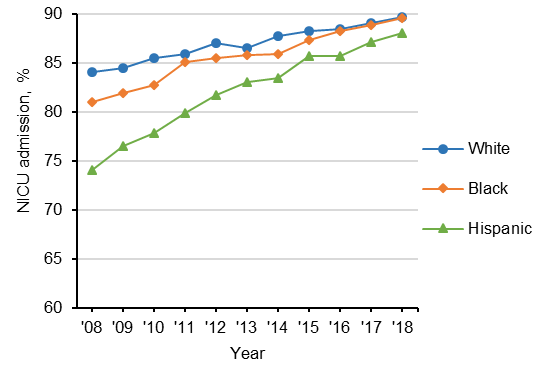

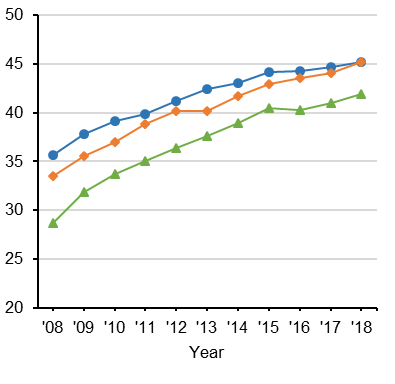


C. Gestational Age ≥37 wk D. All Gestational Age: 2008 vs 2018


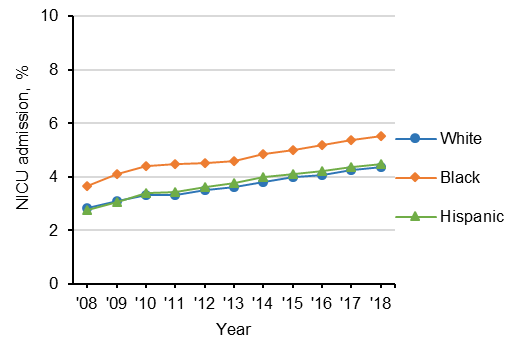

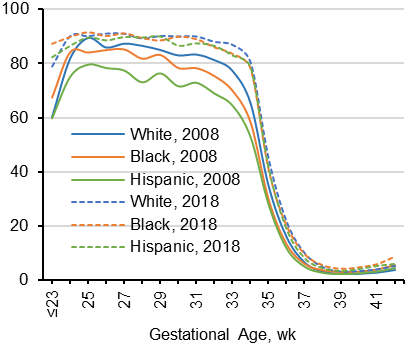


**Supplemental Table 5. Comparison of the Study Population and Total U.S. Birth Cohorts for 2008-2015***

|  | **Study Population** | **United States** |
| --- | --- | --- |
| No. of Births | 26,912,694 | 32,249,785 |
| **Maternal Characteristics, %** | | |
| Race/Ethnicity |  |  |
| White | 53.1 | 53.8 |
| Black | 14.3 | 14.7 |
| Hispanic | 24.8 | 23.6 |
| Other | 7.9 | 7.9 |
| Maternal Age, yr |  |  |
| <20 | 8.0 | 8.2 |
| 20-24 | 23.2 | 23.2 |
| 25-29 | 28.6 | 28.5 |
| 30-34 | 25.3 | 25.1 |
| 35-39 | 12.1 | 12.1 |
| 40-54 | 2.9 | 2.9 |
| Unmarried | 40.6 | 40.6 |
| **Neonatal Characteristics, %** | | |
| Female | 48.8 | 48.8 |
| Multiple gestations | 3.4 | 3.5 |
| Cesarean delivery | 32.5 | 32.5 |
| Birthweight category, g |  |  |
| <1500 | 1.4 | 1.4 |
| 1500-2499 | 6.6 | 6.7 |
| 2500-3999 | 84.1 | 84.0 |
| ≥4000 | 7.8 | 7.8 |
| Gestational age, wk |  |  |
| <32 | 1.6 | 1.6 |
| 32-36 | 8.2 | 8.2 |
| 37-38 | 26.3 | 26.4 |
| 39-40 | 57.1 | 56.9 |
| ≥41 | 6.7 | 6.7 |
| 5-Minute Apgar score |  |  |
| <7 | 2.0 | 1.9 |
| 7-8 | 12.9 | 12.0 |
| 9-10 | 84.6 | 85.6 |

*Because most of the excluded infants had birth information recorded using the earlier 1989 version of the U.S. birth certificate (up to 2015), we assessed the representativeness of our study cohort by comparing birth cohorts recorded with the 2003 revision to total U.S. birth cohorts for 2008-2015. Starting with 2016, the 2003 revision had been implemented in all U.S. states and the District of Columbia.

**Supplemental Table 6. Temporal Trends for Crude NICU Admission Rates for 2008-2018 by Race/Ethnicity, All Birth Weights Limited to 27 States***

|  | NICU Admission, % (95% CI) | | | |
| --- | --- | --- | --- | --- |
| Year | All | White | Black | Hispanic |
| 2008 | 6.62 (6.59-6.65) | 6.58 (6.54-6.62) | 9.09 (8.99-9.18) | 5.70 (5.65-5.75) |
| 2009 | 7.03 (7.00-7.06) | 6.85 (6.80-6.89) | 9.74 (9.65-9.84) | 6.22 (6.16-6.27) |
| 2010 | 7.36 (7.33-7.39) | 7.11 (7.07-7.15) | 10.21 (10.11-10.31) | 6.56 (6.50-6.62) |
| 2011 | 7.43 (7.40-7.46) | 7.12 (7.07-7.16) | 10.28 (10.18-10.39) | 6.72 (6.66-6.78) |
| 2012 | 7.75 (7.71-7.78) | 7.42 (7.38-7.47) | 10.65 (10.54-10.75) | 7.07 (7.01-7.13) |
| 2013 | 7.96 (7.93-7.99) | 7.65 (7.60-7.69) | 10.63 (10.52-10.73) | 7.33 (7.27-7.39) |
| 2014 | 8.27 (8.24-8.31) | 7.92 (7.87-7.96) | 11.07 (10.97-11.18) | 7.67 (7.60-7.73) |
| 2015 | 8.49 (8.46-8.52) | 8.12 (8.07-8.16) | 11.30 (11.20-11.41) | 7.92 (7.85-7.98) |
| 2016 | 8.71 (8.67-8.74) | 8.31 (8.26-8.35) | 11.72 (11.61-11.82) | 8.09 (8.02-8.15) |
| 2017 | 8.97 (8.93-9.00) | 8.52 (8.47-8.57) | 12.04 (11.94-12.15) | 8.39 (8.32-8.45) |
| 2018 | 9.17 (9.13-9.20) | 8.65 (8.60-8.70) | 12.35 (12.24-12.46) | 8.67 (8.60-8.74) |

*The 27 states that had implemented the revised birth certificate as of January 1, 2008, were the following: California, Colorado, Delaware, Florida, Georgia, Idaho, Indiana, Iowa, Kansas, Kentucky, Michigan, Montana, Nebraska, New Hampshire, New Mexico, New York (including New York City), North Dakota, Ohio, Oregon, Pennsylvania, South Carolina, South Dakota, Tennessee, Texas, Vermont, Washington, and Wyoming. These states represent 65 % of the births to U.S. residents in 2008.

**Reference**

1. Patrick SW, Schumacher RE, Davis MM. Methods of mortality risk adjustment in the NICU: a 20-year review. *Pediatrics*. 2013;131 Suppl 1:S68-S74. doi:10.1542/peds.2012-1427h
2. Richardson DK, Corcoran JD, Escobar GJ, Lee SK. SNAP-II and SNAPPE-II: Simplified newborn illness severity and mortality risk scores. *J Pediatr*. 2001;138(1):92-100. doi:10.1067/mpd.2001.109608
3. Parry G, Tucker J, Tarnow-Mordi W; UK Neonatal Staffing Study Collaborative Group. CRIB II: an update of the clinical risk index for babies score. *Lancet*. 2003;361(9371):1789-1791. doi:10.1016/S0140-6736(03)13397-1
4. Zupancic JA, Richardson DK, Horbar JD, et al. Revalidation of the Score for Neonatal Acute Physiology in the Vermont Oxford Network. *Pediatrics*. 2007;119(1):e156-e163. doi:10.1542/peds.2005-2957
5. Tyson JE, Parikh NA, Langer J, Green C, Higgins RD; National Institute of Child Health and Human Development Neonatal Research Network. Intensive care for extreme prematurity--moving beyond gestational age. *N Engl J Med*. 2008;358(16):1672-1681. doi:10.1056/NEJMoa073059
6. Hosmer Jr DW, Lemeshow S, Sturdivant RX. *Applied logistic regression*. New York, NY: John Wiley & Sons; 2013.
